# Reprogramming the immunosuppressive microenvironment in MSS/pMMR colorectal cancer via synergistic pyroptosis induction and PD-L1 suppression

**DOI:** 10.3389/fimmu.2026.1831497

**Published:** 2026-07-14

**Authors:** Jingyi Mo, Weirui Huang, Rongrong Li, Yiqiong Xie, Lei Chang

**Affiliations:** 1 Nanjing Medical University, Nanjing, Jiangsu, China; 2 School of Pharmacy, Faculty of Medicine, Macau University of Science and Technology, Taipa, Macao SAR, China; 3 Department of Pharmacy, Taikang Xianlin Drum Tower Hospital, Nanjing, Jiangsu, China; 4 Department of Pharmacy, Nanjing Drum Tower Hospital Clinical College of Nanjing University of Chinese Medicine, Nanjing, Jiangsu, China; 5 Department of Pharmacy, Women and Children’s Hospital Affiliated to Ningbo University, Ningbo, Zhejiang, China; 6 Department of Pharmacology, School of Pharmacy, Nanjing Medical University, Nanjing, China

**Keywords:** colorectal cancer, ginsenoside Rg3, liposome, PD-L1, pyroptosis

## Abstract

Colorectal cancer (CRC) with microsatellite-stable (MSS)/pMMR status resists immune checkpoint blockade due to its immunologically “cold” tumor microenvironment. We developed Lipo-LPS-Rg3, a dual-functional nanoliposome co-delivering lipopolysaccharide (LPS) and ginsenoside Rg3, to simultaneously ignite pyroptosis and attenuate PD-L1-associated immune suppression. LPS triggered GSDMD-mediated pyroptosis, releasing DAMPs and recruiting CD8^+^ T cells, while Rg3 reduced PD-L1 expression *in vitro*, at least partly by inhibiting NFATc1 nuclear translocation. *In vivo*, Lipo-LPS-Rg3 decreased PD-L1 protein expression in tumor tissues and promoted both CD8^+^ T-cell infiltration and IFN-γ-producing effector function. The nanoplatform achieved tumor-targeted delivery, induced near-complete regression in colorectal cancer, and exhibited minimal systemic toxicity. By converting “cold” tumors into “hot” and supporting antitumor T-cell activity, Lipo-LPS-Rg3 offers a promising strategy for MSS/pMMR CRC immunotherapy.

## 1 Introduction

Colorectal cancer (CRC) remains a leading cause of global cancer mortality, with the microsatellite-stable/proficient mismatch repair (MSS/pMMR) subtype accounting for ~85% of cases (1, 2). Unlike their MSI-H counterparts, MSS/pMMR tumors are notoriously refractory to immune checkpoint inhibitors (ICIs), owing to an immunologically “cold” tumor microenvironment (TME)-marked by scant T-cell infiltration and persistent PD-L1-mediated immune suppression (3–6). Accumulating evidence implicates hyperactivation of the STAT3/NFATc1 axis as a key driver of constitutive PD-L1 expression in this setting, establishing it as a compelling therapeutic node (7–9). Yet, merely blocking PD-L1 is insufficient without concomitant generation of tumor immunogenicity-a dual challenge that has stymied progress in MSS CRC immunotherapy (10, 11).

**Scheme:**
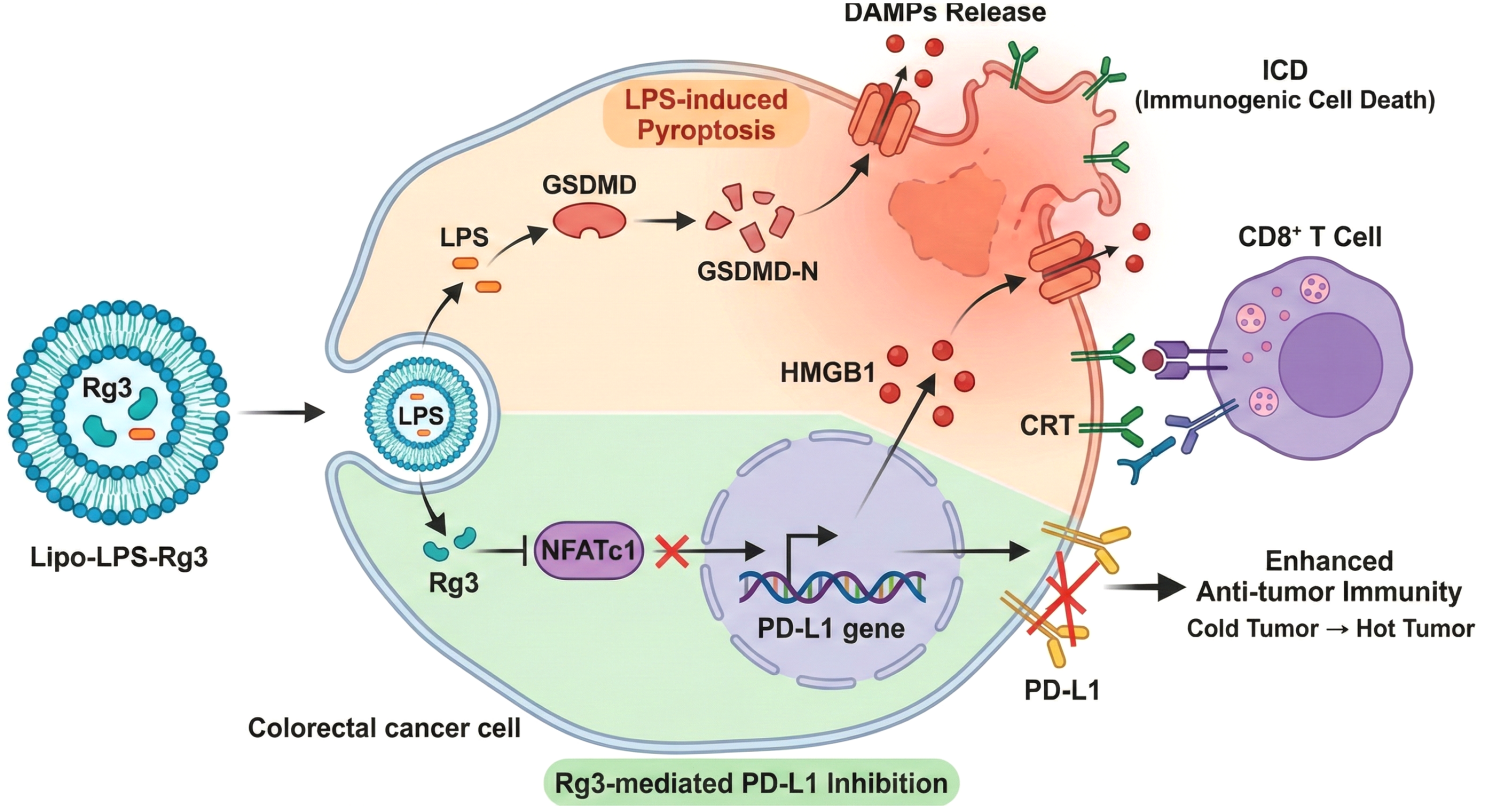
Schematic of Lipo-LPS-Rg3 nanoliposomes for dual modulation of the tumor immune microenvironment. LPS induces pyroptosis and DAMP release to recruit CD8^+^ T cells, while Rg3 suppresses PD-L1 via NFATc1 inhibition, converting “cold” MSS/pMMR tumors into “hot,” immunoresponsive niches.

Ginsenoside Rg3, a natural triterpenoid saponin, has emerged as a promising immunomodulator capable of disrupting PD-L1/PD-1 engagement and suppressing PD-L1 transcription (12, 13). However, its clinical utility is severely limited by poor aqueous solubility and low bioavailability, which hinder effective intratumoral delivery (14, 15). Meanwhile, pyroptosis-a lytic, pro-inflammatory form of programmed cell death-has gained traction as a potent strategy to convert immunologically inert tumors into “hot” niches (16, 17). Lipopolysaccharide (LPS), a canonical pyroptosis inducer, activates non-canonical inflammasomes to cleave Gasdermin D (GSDMD), triggering the release of damage-associated molecular patterns (DAMPs) such as HMGB1 and calreticulin that recruit and activate dendritic cells and CD8^+^ T cells (18–23). Despite its immunostimulatory potential, systemic administration of free LPS is precluded by life-threatening inflammatory toxicity (24, 25).

To surmount these intertwined barriers, we engineered a tumor-targeted nanoliposome, Lipo-LPS-Rg3, for the co-delivery of Rg3 and LPS. We hypothesized that spatially coordinated release of both agents within the TME would achieve synergistic immune reprogramming: LPS-driven pyroptosis would serve as an endogenous “danger signal” to recruit effector T cells, while Rg3 would concurrently dismantle the PD-L1 immune checkpoint by inhibiting NFATc1 nuclear translocation-thereby preserving T-cell functionality (**Scheme**). This dual-action nanoplatform represents not merely a drug delivery system, but a rational immune-engineering strategy designed to overcome the twin pillars of resistance in MSS/pMMR CRC: immune ignorance and immune suppression.

## 2 Materials and methods

### 2.1 Materials

Ginsenoside Rg3 was purchased from Shanghai Yuanye Bio-Technology Co., Ltd. (Shanghai, China). Egg yolk lecithin (SPC) was obtained from AVT (Shanghai) Pharmaceutical Tech Co., Ltd. Lipopolysaccharide (LPS) was purchased from Hefei Baisha Biotechnology Co., Ltd. Coumarin-6 (C6) and IR780 were acquired from Shanghai Yuanye Biotechnology. Cell culture reagents, including RPMI 1640 medium, fetal bovine serum (FBS), and penicillin-streptomycin, were obtained from Nanjing SenBeijia Biological Technology Co., Ltd. Primary antibodies against beta-actin, Lamin B, GSDMD, PD-L1, NFATc1, and secondary antibodies were purchased from Proteintech (Wuhan, China). Antibodies for CRT and HMGB1 were obtained from Abcam (UK). All other chemicals were of analytical grade.

### 2.2 Cell lines and animal

The murine colorectal cancer cell line CT26 was obtained from the Cell Bank of the Chinese Academy of Sciences. Cells were cultured in RPMI 1640 supplemented with 10% FBS and 1% penicillin-streptomycin at 37 °C in a 5% CO_2_ incubator. Male BALB/c mice (6 weeks old) were purchased from Beijing Vital River Laboratory Animal Technology Co., Ltd. All animal experiments were approved by the Animal Ethics Committee of Nanjing Hospital Affiliated to Nanjing Medical University (Approval No. DWSY-24024480).

### 2.3 Preparation and characterization of lipo-LPS-Rg3

#### 2.3.1 Synthesis of nanoliposomes

Lipo-LPS-Rg3 was synthesized via thin-film hydration followed by probe sonication. A lipid film containing egg yolk lecithin and Rg3 was prepared by rotary evaporation and subsequently hydrated with an aqueous LPS solution. The resulting suspension was sonicated (20 W) and purified via ultrafiltration centrifugation (4000 × g) to remove unencapsulated agents. Control liposomes (Lipo-LPS and Lipo-Rg3) were prepared using identical protocols, substituting the respective components with vehicle solvent. For imaging studies, fluorescent liposomes loaded with Coumarin-6 (C6) or IR780 were prepared by incorporating the dye into the organic lipid phase prior to evaporation.

#### 2.3.2 Characterization of liposomes

Particle size, polydispersity index (PDI), and zeta potential were measured using a Nanobrook analyzer (Brookhaven, USA). Morphology was visualized via scanning electron microscopy (SEM) (Quanta 200, FEI, USA) following negative staining with 3% phosphotungstic acid. The encapsulation efficiency of Rg3 was determined via HPLC (Waters 2695) using a TPA-C18 column. Stability was assessed by monitoring particle size over 7 days at 4 °C.

#### 2.3.3 Determination of LPS encapsulation efficiency and drug loading

The encapsulation efficiency (EE%) and drug loading (DL%) of LPS were determined using the phenol-sulfuric acid method. A standard curve was established by reacting glucose standards (2.0 mL aqueous volume) with 6% phenol (1.0 mL) and concentrated sulfuric acid (5.0 mL), followed by absorbance measurement at 490 nm. Lipo-LPS-Rg3 was centrifuged in ultrafiltration tubes (4,000 × g, 30 min) to separate free and encapsulated LPS. To normalize the reaction volume, 1.0 mL of each sample was supplemented with 1.0 mL of distilled water and reacted identically. Blank Lipo-Rg3 was processed under the same conditions to subtract the lipid-induced background absorption. The corrected absorbance values were then fitted to the standard curve to calculate the absolute LPS mass, yielding the final EE% and DL%.

### 2.4 *In vitro* cellular uptake

CT26 cells were seeded in 12-well plates (5×10^4^ cells/well) and incubated overnight. Cells were treated with free C6, Lipo-C6, or Lipo-LPS-Rg3-C6 (1 μg/mL C6 equivalent). Following incubation for 2, 6, and 8 h, cells were harvested, and intracellular fluorescence was quantified via flow cytometry (Accuri C6 Plus, BD, USA).

### 2.5 Assessment of pyroptosis induction

CT26 cells were incubated with PBS, free LPS (20 μg/mL), or Lipo-LPS (20 μg/mL equivalent) for 24 h. The induction of pyroptosis was evaluated by quantifying lactate dehydrogenase (LDH) release in the culture supernatant using a commercial cytotoxicity assay kit according to the manufacturer’s protocol.

### 2.6 Immunofluorescence analysis of DAMPs

To characterize immunogenic cell death (ICD), the expression and localization of damage-associated molecular patterns (DAMPs) were assessed. Following treatment, cells were subjected to immunofluorescence staining for cell surface Calreticulin (CRT) and HMGB1 release. Images were acquired and analyzed using a high-throughput Thunder imaging system (Leica, Germany).

### 2.7 Quantitative real-time PCR

Total RNA was extracted using Trizol reagent and reverse-transcribed into cDNA. qRT-PCR was performed using SYBR Green Master Mix on a LightCycler 480 II (Roche, Germany). Relative gene expression of PD-L1 was calculated using the 2^^-ΔΔCt^ method, normalized to GAPDH. The specific primer sequences are listed in **Table 1**.

**Table 1:** Primer sequences used for qRT-PCR.

Genes		Sequence
GAPDH	forward	5’-AGGTCGGTGTGAACGGATTTG-3’
	reverse	5’-TGTAGACCATGTAGTTGAGGTCA-3’
PD-L1	forward	5’-GCTCCAAAGGACTTGTACGTG-3’
	reverse	5’-TGATCTGAAGGGCAGCATTTC-3’

### 2.8 Western blot analysis

For *in vitro* studies, cells were treated with Rg3 (30 μM) or Lipo-Rg3 for 24 h. For *in vivo* studies, tumor tissues were homogenized in RIPA lysis buffer containing PMSF. Total protein and nuclear fractions were extracted and quantified via BCA assay. Proteins were resolved by SDS-PAGE, transferred to PVDF membranes, and incubated with primary antibodies against GSDMD, GSDMD-N, PD-L1, and NFATc1 at 4 °C overnight. β-actin and Lamin B served as internal controls.

### 2.9 *In vitro* co-culture and T cell activation assay

To evaluate Th1 polarization, purified splenic T cells were isolated from healthy BALB/c mice using a 70-μm strainer and RBC lysis buffer. These T cells were then co-cultured with CT26 cells that had been pre-treated with PBS, Lipo-Rg3, Lipo-LPS, or Lipo-LPS-Rg3 for 24 h. During the final hours of co-culture, T cells were stimulated with a cocktail containing PMA, ionomycin, and protein transport inhibitors. Harvested T cells were first surface-stained with a PE-conjugated anti-CD4 antibody at 4 °C. Subsequently, cells were fixed, permeabilized, and intracellularly stained with a FITC-conjugated anti-IFN-γ antibody. All specific flow cytometry antibodies were sourced from Elabscience (Wuhan, China). Finally, data were acquired using a CytoFLEX flow cytometer (Beckman Coulter, USA) to quantify the proportion of IFN-γ^+^ CD4^+^ Th1 cells.

### 2.10 *In vivo* biodistribution

Tumor-bearing mice were established by subcutaneous injection of CT26 cells (1× 10^6^). When tumors reached a suitable size, mice received an intravenous injection of IR780 or Lipo-IR780 (1 mg/kg). Fluorescence distribution was monitored at 0, 4, 8, 12, 24, and 48 h using an IVIS Spectrum system (PerkinElmer, USA). Major organs were harvested at the endpoint for ex vivo imaging.

### 2.11 *In vivo* anti-tumor efficacy

CT26 tumor-bearing mice were randomized into four groups (n=3): Control, Lipo-Rg3, Lipo-LPS, and Lipo-LPS-Rg3. Treatments were administered intravenously every other day for five doses (LPS: 2 mg/kg; Rg3: 30 mg/kg). Tumor volume (V = ab^2^/2) and body weight were recorded every two days. On day 14, mice were sacrificed, and tumors/organs were harvested for further analysis.

### 2.12 Histological and immunofluorescence analysis

Tumor tissues were fixed in 4% paraformaldehyde, embedded in paraffin, and sectioned. Histopathological changes and apoptosis were evaluated via Hematoxylin and Eosin (H&E) staining and TUNEL assay, respectively. Additionally, immunofluorescence staining was performed to visualize tumor-infiltrating lymphocytes (CD4^+^, CD8^+^) and quantify PD-L1 expression levels.

### 2.13 *In vivo* T-cell flow cytometry

Tumor tissues were harvested at the end of treatment and mechanically minced into small fragments. The tissues were digested in serum-free medium containing collagenase and DNase I to obtain single-cell suspensions. After filtration through a 70-μm cell strainer, cells were washed with PBS and resuspended in complete medium. For intracellular cytokine detection, tumor-derived single-cell suspensions were stimulated with PMA for 4–6 h at 37 °C in a 5% CO_2_ incubator. A protein transport inhibitor was added during stimulation to promote intracellular accumulation of IFN-γ. After stimulation, cells were collected and stained with a fixable viability dye, followed by surface staining with anti-CD45, anti-CD3, and anti-CD8 antibodies. Cells were then fixed, permeabilized, and stained intracellularly with anti-IFN-γ antibody according to the manufacturer’s instructions. Flow cytometric data were acquired using a flow cytometer and analyzed with FlowJo software.

### 2.14 *In vivo* biosafety evaluation

To assess systemic toxicity, healthy mice (n=3) were randomized into four groups and administered saline, Lipo-LPS, Lipo-Rg3, or Lipo-LPS-Rg3 via tail vein injection every other day for four doses. Body weights were monitored throughout the treatment regimen. On day 8, mice were sacrificed to collect serum and major organs. Biochemical parameters, including alanine aminotransferase (ALT), aspartate aminotransferase (AST), creatinine (CREA), blood urea nitrogen (BUN), and C-reactive protein (CRP), were quantified. Additionally, major organs were subjected to H&E staining to detect potential histopathological damage.

### 2.15 Statistical analysis

Data were analyzed using GraphPad Prism and Origin software. Results are presented as mean pm standard deviation (SD). Statistical significance was determined using Student’s t-test or one-way ANOVA. *P* < 0.05 was considered statistically significant (*P < 0.05, ** *P* < 0.01, *** *P* < 0.001, **** *P* < 0.0001).

## 3 Results

### 3.1 Preparation, characterization, and cellular internalization of lipo-LPS-Rg3

**Figure 1:**
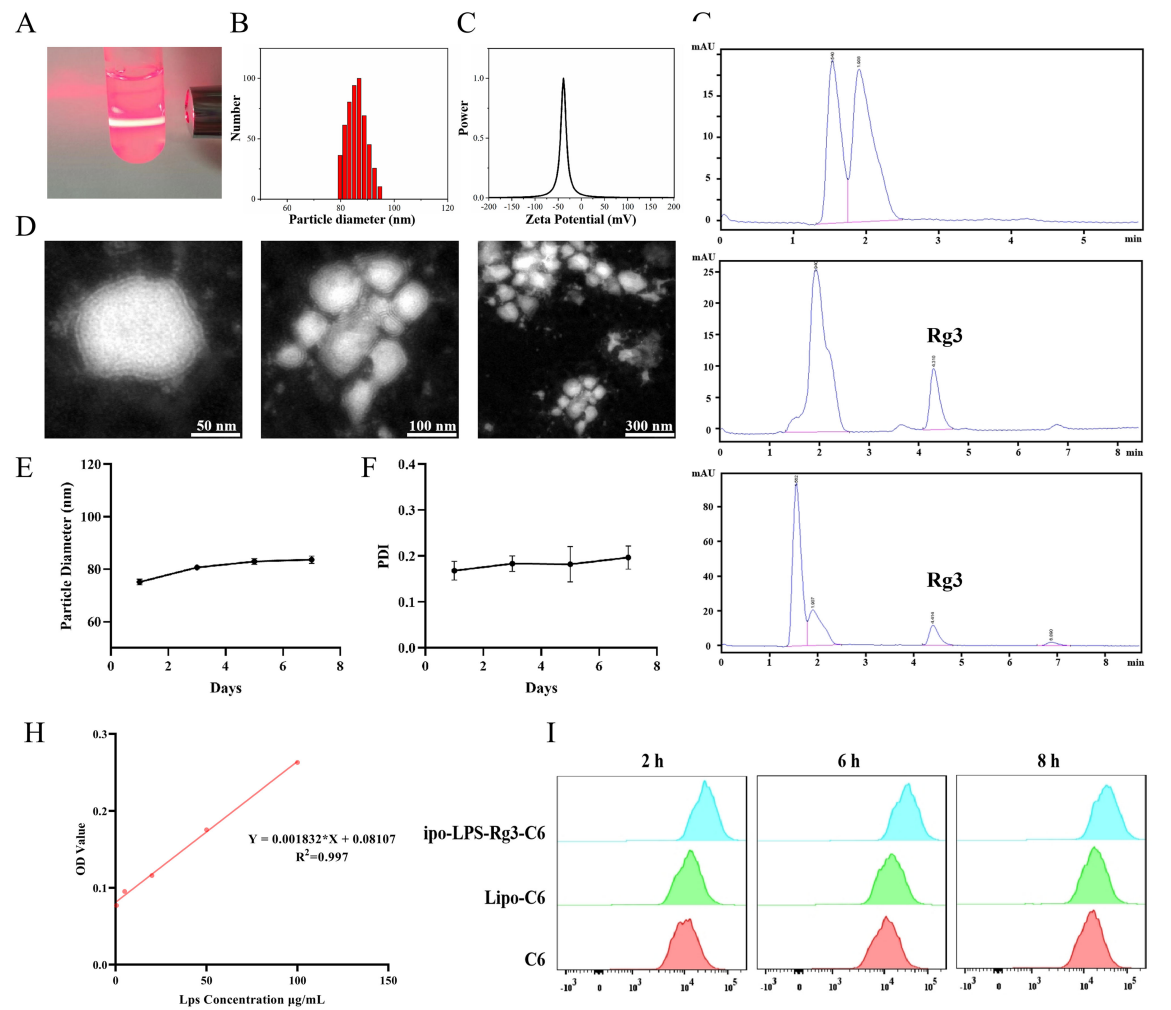
Preparation and physicochemical characterization of lipo-LPS-Rg3. **(A)** Visual appearance of lipo-LPS-Rg3 solution showing the Tyndall effect under laser irradiation. **(B)** Representative particle size distribution and **(C)** Zeta potential distribution measured by DLS. **(D)** Representative SEM images of Lipo-LPS-Rg3 at different magnifications. Scale bars: 50 nm, 100 nm, 300 nm. **(E, F)** Stability of lipo-LPS-Rg3 stored at 4 °C for 7 days, monitoring changes in particle diameter **(E)** and PDI **(F)**. **(G)** HPLC chromatograms of demulsified lipo-LPS (blank), Rg3 standard, and demulsified lipo-LPS-Rg3. **(H)** Standard calibration curve for LPS quantification (phenol-sulfuric acid method. **(I)** Flow cytometry histograms of CT26 cells incubated with free C6, Lipo-C6, and lipo-LPS-Rg3-C6 for 2, 6, and 8 h. Data are presented as mean ± SD.

To overcome the poor solubility of ginsenoside Rg3 and the systemic toxicity of lipopolysaccharide (LPS), while enabling their co-delivery for synergistic antitumor therapy, we developed a nanoliposomal formulation-Lipo-LPS-Rg3. This platform aims to enhance tumor accumulation via the EPR effect and ensure stable, efficient drug loading. The Lipo-LPS-Rg3 nanoliposomes were successfully synthesized via thin-film hydration. Visual inspection revealed a clear, transparent solution exhibiting a distinct Tyndall effect under laser irradiation, confirming the formation of colloidal nanostructures (**Figure 1A**). DLS analysis demonstrated that the Lipo-LPS-Rg3 possessed an average hydrodynamic diameter of 74.83 ± 1.22 nm (**Figure 1B**). This size range (1–100 nm) is optimal for deep tissue penetration and accumulation via the EPR effect. ref The PDI was determined to be 0.19 ± 0.045, indicating a uniform particle distribution. Furthermore, the zeta potential was measured at -33.41 ± 3.90 mV (**Figure 1C**), suggesting sufficient electrostatic repulsion to maintain colloidal stability ref. Morphological analysis via SEM corroborated the DLS data, showing that the liposomes maintained a spherical, intact lipid bilayer structure with consistent diameters (**Figure 1D**). To evaluate the feasibility of storage and potential clinical translation, we assessed the physicochemical stability of Lipo-LPS-Rg3 under refrigerated conditions. Stability studies demonstrated that Lipo-LPS-Rg3 retained structural integrity over 7 days at 4 °C, with negligible changes in particle size or PDI (**Figures 1E, F**). Furthermore, the formulation achieved excellent dual-drug loading capacity. HPLC analysis confirmed an EE% of 73.97% and a DL% of 12.97% for Rg3, demonstrating high method specificity with a sharp peak at the retention time of 4.4 min (**Figure 1G**), while the phenol-sulfuric acid method demonstrated an EE% of 84.53% and a DL% of 12.59% for LPS (**Figure 1H**).

Following physicochemical characterization, we investigated the cellular internalization capability of the Lipo-LPS-Rg3, which is a prerequisite for their biological function. Using C6 as a fluorescent probe, flow cytometric analysis revealed a time-dependent increase in fluorescence intensity in CT26 cells treated with Lipo-LPS-Rg3-C6 compared to free C6 and Lipo-C6 (2, 6, and 8 h) (**Figure 1I**). Notably, the Lipo-LPS-Rg3-C6 group exhibited the highest fluorescence intensity. This superior internalization is likely attributable to the structural similarity between Ginsenoside Rg3 and cholesterol; Rg3 incorporation may enhance the affinity of the liposomal membrane for the cell surface, thereby facilitating membrane fusion and endocytosis ref. Collectively, these data indicate that Lipo-LPS-Rg3 possesses the requisite physicochemical properties and membrane affinity for effective intracellular drug delivery.

### 3.2 Dual-action nanotherapy converts immunosuppressive “cold” tumors into immunogenic “hot” niches via pyroptosis induction and PD-L1 suppression

A major barrier to effective immunotherapy in microsatellite-stable (MSS) colorectal cancer is the immunologically “cold” tumor microenvironment-characterized by low immunogenicity and persistent immune checkpoint expression, particularly PD-L1 (3, 26). To address this dual challenge, our Lipo-LPS-Rg3 nanoplatform was rationally designed to simultaneously (i) trigger immunogenic pyroptotic cell death and (ii) suppress PD-L1-mediated immune evasion. Here, we provide mechanistic evidence that this dual-action strategy successfully reprograms the tumor immune landscape.

**Figure 2:**
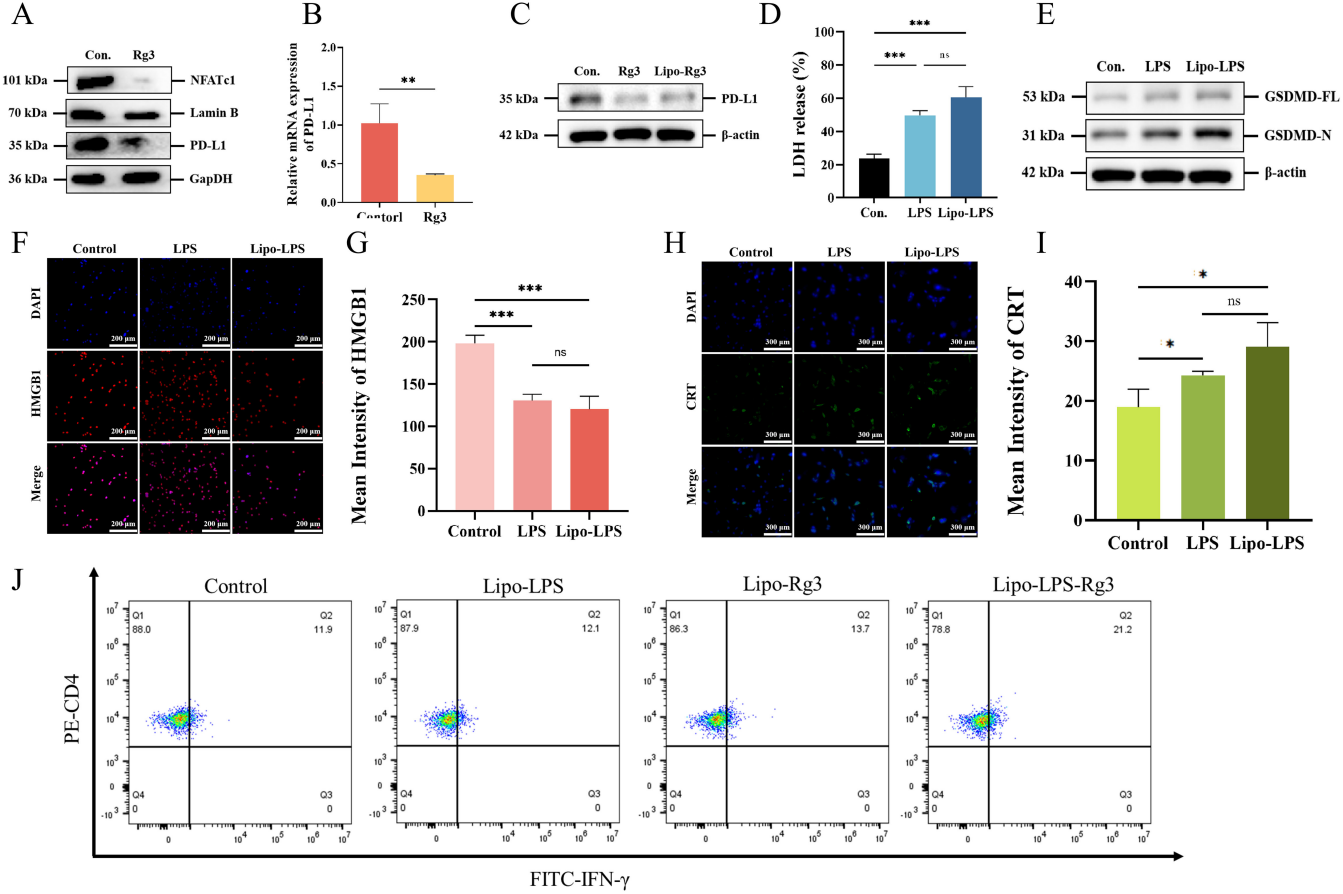
Lipo-LPS-Rg3 downregulates PD-L1 expression and induces pyroptosis *in vitro*. **(A)** Western blot analysis of nuclear NFATc1 and total PD-L1 expression in CT26 cells treated with Rg3. Lamin B and GAPDH served as loading controls for nuclear and total protein, respectively. **(B)** Relative mRNA expression levels of PD-L1 in CT26 cells normalized to GAPDH (**P < 0.01). **(C)** Western blot analysis of PD-L1 expression in cells treated with lipo-Rg3. **(D)** LDH release assay indicating plasma membrane rupture in LPS and lipo-LPS treated cells compared to control (**P < 0.001). **(E)** Western blot analysis of GSDMD-FL and cleaved GSDMD-N expression in CT26 cells. **(F–I)** Evaluation of immunogenic cell death markers. Representative immunofluorescence images of HMGB1 nuclear efflux **(F)** and Calreticulin (CRT) membrane exposure **(H)**, with corresponding semi-quantitative analysis of HMGB1 **(G)** and CRT **(I)** fluorescence intensity. Scale bars: 200 μm for HMGB1 and 300 μm for CRT. (*P < 0.05, ***P < 0.001, ns, not significant). **(J)** Flow cytometry analysis of CD4^+^ T cell activation and Th1 polarization *in vitro* following lipo-LPS-Rg3 treatment.

First, we examined the impact of Rg3 on PD-L1 regulation in murine CT26 colon carcinoma cells-a representative MSS/pMMR model with high baseline PD-L1 expression. Both free Rg3 and liposomal Rg3 (Lipo-Rg3) significantly suppressed PD-L1 at the protein and mRNA levels (**Figures 2A, B**), confirming that nanoencapsulation does not compromise Rg3’s bioactivity. Importantly, mechanistic investigation revealed that Rg3 inhibited the nuclear translocation of NFATc1 (**Figure 2C**), a transcription factor recently implicated in constitutive PD-L1 upregulation in CRC. These findings suggest that Rg3-mediated inhibition of NFATc1 signaling may contribute to PD-L1 downregulation in CT26 cells.

In parallel, we assessed whether the LPS payload could ignite an immunogenic cascade through pyroptosis-a lytic, pro-inflammatory form of programmed cell death known to release damage-associated molecular patterns (DAMPs). Treatment with Lipo-LPS induced robust LDH release (**Figure 2D**), a hallmark of plasma membrane rupture during pyroptosis. This was corroborated by marked cleavage of gasdermin D (GSDMD) to its active N-terminal fragment (GSDMD-N), the executor of pyroptotic pore formation (**Figure 2E**). Critically, immunofluorescence imaging confirmed the extracellular exposure of two canonical DAMPs: HMGB1 translocated from the nucleus to the cytoplasm and extracellular space, while calreticulin (CRT) was prominently displayed on the cell surface (**Figures 2F–I**)-both serving as “eat-me” signals for dendritic cell recruitment and cross-priming of T cells.

To elucidate the direct immunological consequences of these tumor-intrinsic modulations on effector T cell activation, we further evaluated the functional state of CD4^+^ T cells in an *in vitro* co-culture system. Flow cytometric analysis revealed that while single-agent treatments elicited marginal improvements in T cell activation (12.1% for Lipo-LPS and 13.7% for Lipo-Rg3), the dual-loaded Lipo-LPS-Rg3 formulation dramatically bolstered the proportion of IFN-γ producing Th1 cells to 21.2%, compared to 11.9% in the untreated control (**Figure 2J**). This polarization highlights that the DAMPs released via pyroptosis provide critical co-stimulatory signals, which, when synergized with the alleviation of PD-L1-mediated immune suppression, potently awaken the cytolytic potential of CD4^+^ T cells.

Together, these results demonstrate that Lipo-LPS-Rg3 functions as a dual-modulator: it reduces PD-L1-associated immune inhibitory signaling while converting dying tumor cells into endogenous immunogenic stimuli via pyroptosis-driven DAMP release. This coordinated action-simultaneously attenuating tumor immune evasion and amplifying immune activation-lays the foundation for transforming immunologically inert tumors into T-cell-inflamed, “hot” microenvironments amenable to checkpoint blockade or adoptive immunotherapy.

### 3.3 Lipo-LPS-Rg3 Enables tumor-targeted delivery and synergistically reprograms the immunosuppressive microenvironment to drive potent antitumor immunity

**Figure 3:**
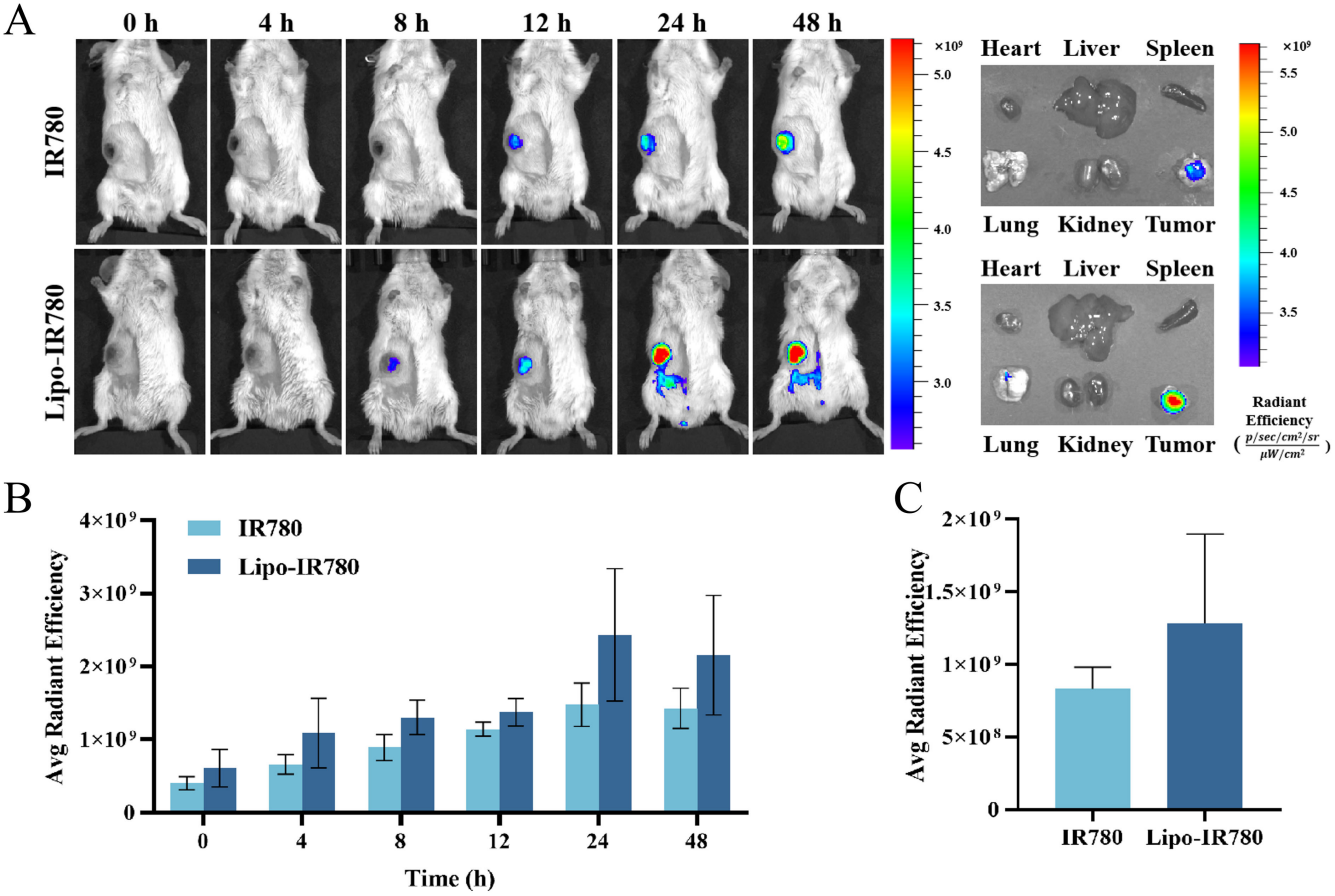
*In vivo* biodistribution and tumor-targeting capability. **(A)** Representative *in vivo* fluorescence images of CT26 tumor-bearing mice at indicated time points (0, 4, 8, 12, 24, 48 h) following intravenous injection of free IR780 or lipo-IR780. **(B)** Quantitative analysis of average radiant efficiency in the tumor region over time. **(C)** *Ex vivo* fluorescence images and quantification of major organs and tumors harvested at 48 h post-injection. Data are presented as mean ± SD (*n* = 3).

Effective tumor targeting is a prerequisite for maximizing therapeutic efficacy while minimizing systemic toxicity-particularly when delivering potent but potentially hazardous agents like LPS (27). To evaluate the *in vivo* biodistribution of our nanoplatform, we loaded liposomes with the near-infrared dye IR780 and administered them to CT26 tumor-bearing mice. Live imaging revealed that Lipo-IR780 accumulated preferentially in tumors, with fluorescence intensity peaking at 24-48 h post-injection-significantly higher and more sustained than free IR780 (**Figures 3A, B**). Ex vivo imaging further confirmed enhanced tumor retention (**Figure 3C**), underscoring the role of the EPR effect in enabling passive tumor targeting. This efficient delivery lays the foundation for localized, high-concentration drug action within the tumor bed. Notably, typical RES (e.g., liver and spleen) accumulation appears visually imperceptible in whole-body imaging. Because the global color scale was normalized to accommodate the extraordinarily high EPR-mediated tumor fluorescence, the comparatively weaker, yet biologically present, RES signals were inadvertently compressed into the invisible range.

**Figure 4:**
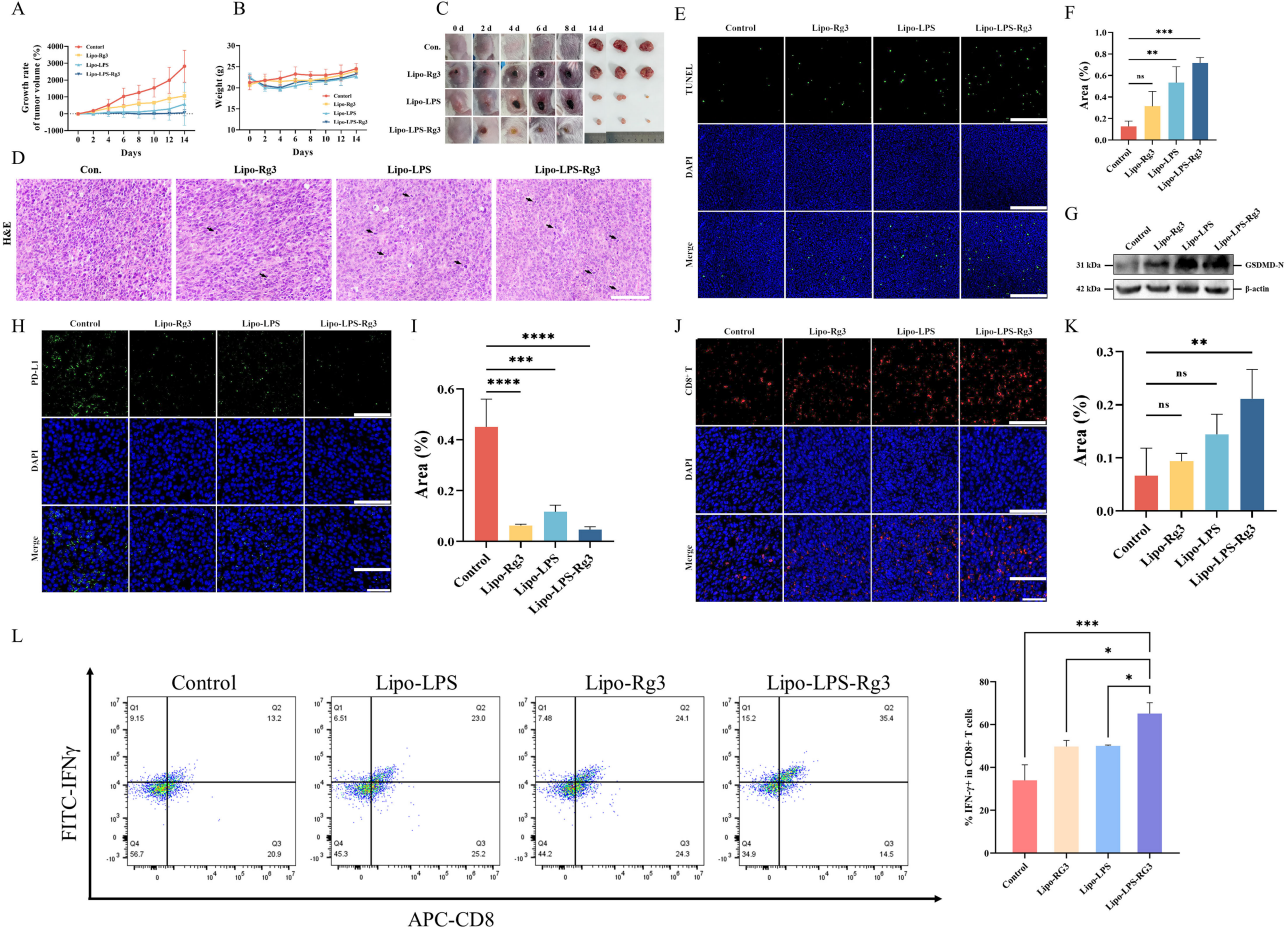
*In vivo* anti-tumor efficacy and immune microenvironment modulation. **(A)** Tumor volume growth curves of CT26 tumor-bearing mice in different treatment groups (n=3). **(B)** Body weight changes of mice monitored during the treatment period. **(C)** Representative macroscopic photographs of tumors showing morphology and surface ulceration over time. **(D)** Representative H&E staining tumor sections showing histological changes. Black arrows indicate necrotic areas. Scale bar: 50 μm. **(E, F)** TUNEL staining of tumor tissues visualizing DNA fragmentation (green) indicative of cell death. Scale bar: 50 μm. **(G)** Western blot analysis of GSDMD-N expression in tumor tissues. **(H–K)** Immunofluorescence staining of PD-L1 (red) **(H, I)** and CD8^+^ T cells (red) **(J, K)** in tumor sections. Nuclei were counterstained with DAPI (blue). **(L)** Representative flow cytometry plots and quantification of IFN-γ^+^ cells among CD8^+^ T cells in tumor single-cell suspensions. Cells were gated on live CD45^+^CD3^+^ T cells, and IFN-γ^+^ cells within the CD8^+^ T-cell population were quantified. Scale bar: 50 μm. Data are presented as mean ± SD. (*P < 0.05, ** *P* < 0.01, *** *P* < 0.001, **** *P* < 0.0001).

We next assessed therapeutic outcomes in a subcutaneous CT26 model-a prototypical “cold” MSS colorectal cancer resistant to conventional immunotherapy. While monotherapies with Lipo-LPS (pyroptosis inducer) or Lipo-Rg3 modestly delayed tumor growth, only the dual-loaded Lipo-LPS-Rg3 elicited profound antitumor responses, with several mice achieving near-complete tumor regression (**Figure 4A**). Importantly, no significant body weight loss was observed, indicating a favorable safety profile (**Figure 4B**).

Notably, tumors treated with LPS-containing formulations developed surface ulceration (**Figure 4C**), a clinical sign suggestive of robust inflammatory activation. Histological analysis by H&E staining revealed extensive cell lysis and nuclear fragmentation (**Figure 4D**), hallmarks of lytic cell death. Further characterization confirmed this as pyroptosis: TUNEL staining showed widespread DNA fragmentation (**Figures 4E, F**), and Western blotting demonstrated marked upregulation of cleaved GSDMD-N specifically in the combination group (**Figure 4G**).

Critically, this pyroptotic burst did not occur in isolation. The resulting release of DAMPs, such as HMGB1 and CRT as shown *in vitro*, appeared to ignite an immune cascade *in vivo*. Immunofluorescence analysis of tumor sections revealed two pivotal changes: (i) a marked reduction in PD-L1 protein expression in tumor tissues (**Figures 4H, I**), which is consistent with our *in vitro* findings suggesting Rg3-associated inhibition of the NFATc1/PD-L1 axis, and (ii) a striking influx of CD8^+^ cytotoxic T lymphocytes into the tumor core (**Figures 4J, K**). To further determine whether these infiltrating CD8^+^ T cells acquired effector function, tumor single-cell suspensions were analyzed by flow cytometry. After gating on live CD45^+^CD3^+^ T cells, the proportion of IFN-γ^+^ cells within the CD8^+^ T-cell population was increased by either Lipo-Rg3 or Lipo-LPS treatment and was further elevated in the Lipo-LPS-Rg3 combination group (**Figure 4L**). These results indicate that the combination therapy not only promotes CD8^+^ T-cell infiltration but also enhances their IFN-γ-producing effector function.

Together, these findings suggest a coordinated antitumor immune remodeling process: LPS-driven pyroptosis converts immunologically inert tumor cells into a source of endogenous danger signals, thereby facilitating T-cell recruitment; meanwhile, Rg3 is associated with reduced PD-L1 expression in tumor tissues, which may help relieve immunosuppressive signaling and support the effector function of infiltrating CD8^+^ T cells. However, direct confirmation of transcriptional regulation of PD-L1 within tumor tissues requires further investigation.

### 3.5 Systemic safety evaluation

**Figure 5:**
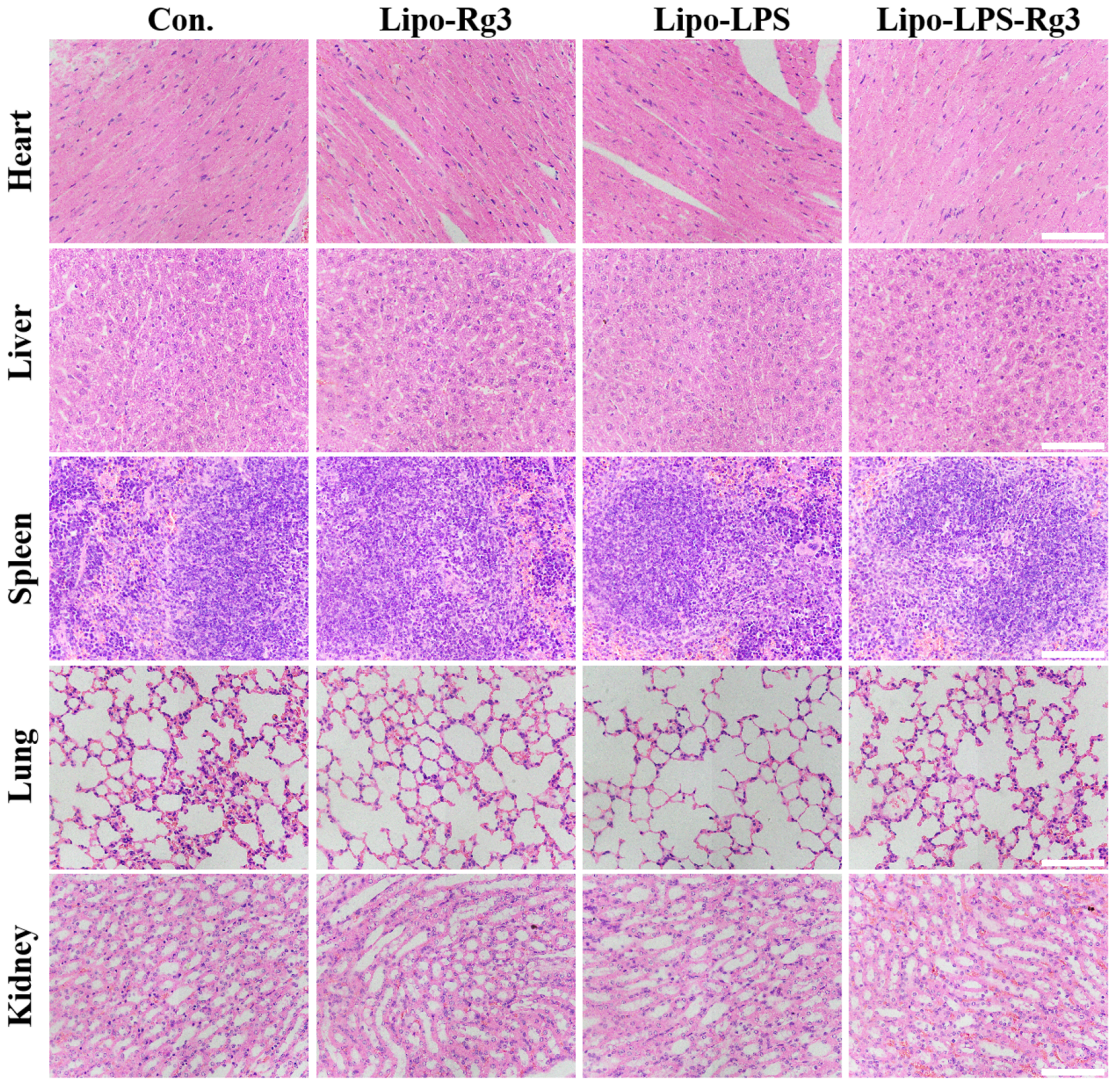
Systemic biosafety evaluation. H&E staining of major organs. Scale bar: 50 μm.

The clinical translation of LPS-based immunotherapies has long been hindered by its potent systemic inflammatory toxicity (28). To evaluate the systemic biosafety of our nanoplatform, healthy mice were monitored following treatment. As expected, LPS-containing formulations induced transient, self-limiting weight loss, but the Lipo-LPS-Rg3 group recovered more rapidly (**Supplementary Figure 1A**). Serum biochemistry revealed no hepatotoxicity (ALT, AST) or severe systemic inflammation across all groups, and notably, Lipo-LPS-Rg3 prevented the mild renal stress (BUN, CREA elevations) induced by Lipo-LPS alone (**Supplementary Figures 1B–E**). Crucially, H&E staining of major organs showed no structural damage or inflammatory infiltration in any group (**Figure 5**). Collectively, Lipo-LPS-Rg3 demonstrates a favorable safety profile, effectively containing LPS toxicity for safe *in vivo* applications.

## 4 Discussion and conclusion

Immunotherapy, particularly immune checkpoint blockade (ICB), has transformed cancer treatment; however, its efficacy in microsatellite-stable (MSS)/proficient mismatch repair (pMMR) colorectal cancer (CRC)-which constitutes ~85% of cases-remains limited due to an immunologically “cold” tumor microenvironment (TME) characterized by low tumor mutational burden, scarce T-cell infiltration, and dominant immune evasion mechanisms (29–34). Overcoming this dual barrier-low immunogenicity and checkpoint-mediated suppression-requires a rationally integrated strategy.

The liposomal delivery platform represents a cornerstone of our therapeutic strategy, offering multiple advantages that are critical for successful tumor immunotherapy. Our nanoliposome enables the simultaneous encapsulation of hydrophilic LPS and hydrophobic Rg3 within a single carrier, ensuring spatial and temporal synchronization of pyroptosis induction and Rg3-associated PD-L1 regulatory effects at the tumor site-a significant advantage over free drug combinations that often suffer from mismatched pharmacokinetic profiles and suboptimal cellular colocalization. With an optimized size, our formulation exploits the Enhanced Permeability and Retention effect for passive tumor targeting, which is particularly crucial for delivering potent immunomodulators like LPS. This targeted delivery confines immunostimulatory activity to the tumor microenvironment while minimizing the risk of systemic inflammatory toxicity, thereby expanding the therapeutic window of potentially hazardous agents. Furthermore, the lipid-based composition provides excellent biocompatibility and biodegradability, ensuring that the observed therapeutic effects stem from the specific payloads rather than vehicle-induced immune activation. Collectively, our Lipo-LPS-Rg3 system demonstrates how rational liposomal engineering can transform potentially hazardous systemic agents into safe, localized, and potent orchestrators of the tumor immune microenvironment, providing a versatile platform for combination immunotherapy.

Within this rationally engineered framework, ginsenoside Rg3, a hydrophobic compound with poor bioavailability, is efficiently solubilized and encapsulated within the lipid bilayer. Once delivered, it reduced PD-L1 expression in CT26 cells *in vitro*, at least partly through inhibition of NFATc1 nuclear translocation-a key transcriptional regulator recently implicated in constitutive PD-L1 upregulation in MSS CRC (13, 35). *In vivo*, immunofluorescence staining further showed decreased PD-L1 protein expression in tumor tissues after Lipo-LPS-Rg3 treatment, which is consistent with, but does not by itself directly prove, transcriptional inhibition of PD-L1 within the tumor tissue. This mechanistic insight extends the known immunomodulatory effects of ginsenosides to a specific signaling axis with direct therapeutic relevance, while indicating that further tissue-level transcript analyses would be required to fully confirm this regulatory mechanism *in vivo*.

Concurrently, liposomal delivery of lipopolysaccharide (LPS) triggered robust GSDMD-dependent pyroptosis, evidenced by membrane rupture, LDH release, and cleavage of GSDMD-N (36–39). Critically, this lytic cell death was highly immunogenic: it induced the extracellular release of HMGB1 and surface exposure of calreticulin-canonical damage-associated molecular patterns (DAMPs) that promote dendritic cell maturation and cross-priming of CD8^+^ T cells (40–43). *In vivo*, this translated into a marked influx of cytotoxic T lymphocytes into previously “cold” tumors, effectively reversing immune exclusion. Consistently, flow cytometric analysis of tumor-derived single-cell suspensions showed that the proportion of IFN-γ^+^ cells within CD8^+^ T cells was increased by Lipo-Rg3 or Lipo-LPS monotherapy and was further enhanced in the Lipo-LPS-Rg3 combination group, indicating that the recruited CD8^+^ T cells acquired stronger effector function rather than merely accumulating within the tumor tissue. The true innovation of Lipo-LPS-Rg3 lies in the spatiotemporal synergy between its two payloads: pyroptosis provides the “spark” to recruit immune cells, while Rg3 is associated with reduced PD-L1 expression and may help relieve PD-L1-mediated immunosuppressive signaling, a combination unattainable with either monotherapy. This dual action culminated in near-complete tumor regression in a resistant CT26 model, far surpassing single-agent effects. Importantly, despite LPS’s notorious systemic toxicity (44), our liposomal formulation exhibited an excellent safety profile, with only transient weight loss and no significant hepatorenal or inflammatory abnormalities. Notably, the Lipo-LPS-Rg3 group showed better tolerability than Lipo-LPS alone, suggesting Rg3 may exert protective effects against LPS-induced stress-an added benefit warranting further investigation.

In summary, Lipo-LPS-Rg3 represents a paradigm-shifting approach for MSS/pMMR CRC: it converts immunologically inert tumors into T-cell-inflamed niches not by external stimulation, but by reprogramming tumor cell fate via pyroptosis and attenuating tumor immune resistance through Rg3-associated PD-L1 suppression. Coupled with its tumor-targeting capability, excellent cargo-loading efficiency, and biocompatibility, this nanoplatform exemplifies the potential of liposome-based delivery systems to safely codeliver antagonistic agents, offering a clinically translatable strategy to unlock immunotherapy responsiveness in “cold” cancers. Nevertheless, direct confirmation of PD-L1 transcriptional regulation in tumor tissues, such as by tumor tissue qPCR, RNAscope, or other *in situ* transcript-level analyses, remains an important direction for future investigation.

## Data Availability

The original contributions presented in the study are included in the article/**Supplementary Material**. Further inquiries can be directed to the corresponding authors.
