# Supplementary material for: Reprogramming the immunosuppressive microenvironment in MSS/pMMR colorectal cancer via synergistic pyroptosis induction and PD-L1 suppression
Source: Front Immunol. 2026 Jul 14;17:1831497. doi: 10.3389/fimmu.2026.1831497 (PMC13407769; doi:10.3389/fimmu.2026.1831497)
Supplement: Supplementary file 1 [file DataSheet1.docx]

**Supplementary materials**

Systemic Physiological and Biochemical Evaluation Mice treated with LPS-containing formulations exhibited transient body weight loss (<15%), likely reflecting an acute but self-limiting inflammatory response. Notably, animals receiving the dual-agent Lipo-LPS-Rg3 recovered more rapidly than those in the Lipo-LPS monotherapy group. Comprehensive serum biochemistry at the study endpoint revealed no evidence of hepatotoxicity (ALT, AST) or severe systemic inflammation (CRP) across all groups. While the Lipo-LPS group showed mild, non-clinical elevations in renal markers (BUN and CREA), these changes were absent in the Lipo-LPS-Rg3 group, which remained comparable to saline controls.


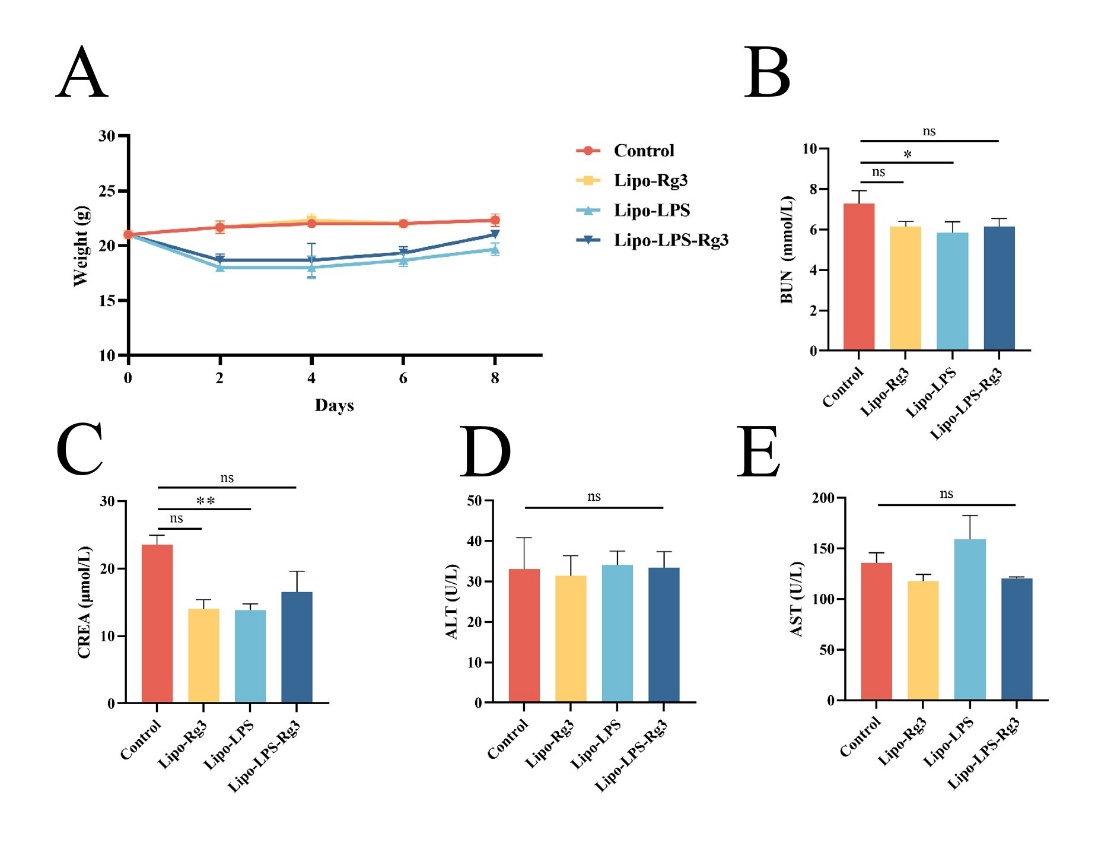


Figure S1. Systemic physiological and biochemical evaluation. (A) Body weight monitoring of healthy mice (n =3). (B-E) Serum levels of (B) BUN, (C) CREA, (D) ALT, and (E) AST on day 8. Data are presented as mean ± SD. (* *P* < 0.05, ** *P* < 0.01, ns: not significant)
